# Improving Rational Treatment of Malaria: Perceptions and Influence of RDTs on Prescribing Behaviour of Health Workers in Southeast Nigeria

**DOI:** 10.1371/journal.pone.0014627

**Published:** 2011-01-31

**Authors:** Benjamin S. C. Uzochukwu, Emmanuel Onwujekwe, Nkoli N. Ezuma, Ogochukwu P. Ezeoke, Miriam O. Ajuba, Florence T. Sibeudu

**Affiliations:** 1 Department of Community Medicine, College of Medicine, University of Nigeria, Enugu-Campus, Enugu, Nigeria; 2 Health Policy Research Group, College of Medicine, University of Nigeria, Enugu-Campus, Enugu, Nigeria; 3 Department of Health Administration and Management, College of Medicine, University of Nigeria, Enugu-Campus, Enugu, Nigeria; 4 Department of Sociology, University of Nigeria, Nsukka-Campus, Nsukka, Nigeria; 5 Department of Community Medicine, Enugu State University of Technology, Enugu, Nigeria; 6 Department of Nursing Sciences, Nnamdi Azikiwe University, Awka, Nigeria; The George Washington University Medical Center, United States of America

## Abstract

**Introduction:**

Developments in rapid diagnostic tests (RDTs) have opened new possibilities for improved remote malaria diagnosis that is independent of microscopic diagnosis. Studies in some settings have tried to assess the influence of RDTs on the prescribing behaviour of health workers, but such information is generally lacking in Nigeria and many parts of sub-Saharan Africa. This study analysed health workers' perceptions of RDTs and their potential influence on their prescribing and treatment practices after their introduction.

**Methods:**

The study was conducted in four health centers in the Enugu East local government of Enugu State, Nigeria. All 32 health workers in the health centers where RDTs were deployed were interviewed by field workers. Information was sought on their perception of symptoms-based, RDT-based, and microscopy-based malaria diagnoses. In addition, prescription analysis was carried out on 400 prescriptions before and 12 months after RDT deployment.

**Results:**

The majority of the health workers perceived RDTs to be more effective for malaria diagnosis than microscopy and clinical diagnosis. They also felt that the benefits of RDTs included increased use of RDTs in the facilities and the tendency to prescribe more Artemisinin-based combination therapies (ACTs) and less chloroquine and SP. Some of the health workers experienced some difficulties in the process of using RDT kits. ACTs were prescribed in 74% of RDT-negative results.

**Conclusions/Significance:**

RDT-supported malaria diagnosis may have led to the overprescription of ACTs, with the drug being prescribed to people with RDT-negative results. However, the prescription of other antimalarial drugs that are not first-line drugs has been reduced. Efforts should be made to encourage health workers to trust RDT results and prescribe ACTs only to those with positive RDT results. In-depth studies are needed to determine why health workers continue to prescribe ACTs in RDT-negative results.

## Introduction

Malaria has remained a major public health problem in Nigeria. It causes more than 50% of the disease burden [1] and almost 50% of all-cause health expenditure [2]. Also, 20% of all hospital admissions, 30% of outpatient visits, and 10% of hospital deaths are attributable to malaria, while half of Nigeria's population is exposed to at least one episode of malaria every year [3]. Worldwide, it kills more than one million people each year; between 20 and 40% of outpatient visits and 10 to 15% of hospital admissions in Africa are attributed to malaria [3]–[5].

Prompt parasitological confirmation by microscopy or with a rapid diagnostic test (RDT) is recommended for all patients with suspected malaria before treatment is started [6], and confirmed cases of uncomplicated *Plasmodium falciparum* malaria should be treated with artemisinin-based combination therapy (ACT). However, the microscopic diagnosis of malaria is time-consuming, labour-intensive, and costly [7], [8]. There is also a lack of reliable microscopy in the majority of peripheral health centers. On the other hand, clinical diagnosis based on malaria symptoms has proven to be unspecific [9]–[11]. These shortcomings of microscopy and clinical diagnosis have favoured the deployment and use of RDTs, which allows diagnosis even in health settings lacking any laboratory facility. RDTs have been found to be cost-effective both in Nigeria and elsewhere [12]–[16], and they generally cost less than a full course of ACT. Therefore, their introduction should not only improve malaria management but should also limit malaria treatment costs [17].

However, new knowledge is needed about health workers' perceptions of RDTs and their potential influence on their malaria treatment practices. This is important because the use of RDTs to diagnose malaria without recourse to laboratory and clinical approaches is a new experience for health workers. Therefore, the nature of health workers' perceptions of the usefulness of RDTs and their influence on their drug prescription patterns will provide useful information for the effective and sustained scaled-up use of RDTs in health centers.

Predictors of health workers' prescribing practice have been explored in a number of developing country settings [18]–[22] where patient (age, complaint), consultation (time of day, duration), and health worker (level of training) factors have been identified as influencing decisions in prescribing antimalarials. These studies have focused on health workers prescribing correct antimalarials to non-severe febrile children. Some authors have also assessed the influence of RDTs on the treatment practices of providers in other settings [17], [23], but studies have not yet sought to identify the quality of these prescriptions, especially after an intervention to improve the rapid diagnosis of malaria by health workers in Nigeria and many parts of sub-Saharan Africa. At this time, ACT had been introduced in Nigeria as a first-line antimalarial drug [1] as a result of extensive resistance to chloroquine and sulphadoxine-pyrimethamine (SP), and following WHO's recommendation that a combination of antimalarials be used to treat malaria caused by P. falciparum [24]. However, chloroquine was still being used by health workers in Nigeria.

This study therefore investigated the antimalarial prescription patterns of health workers before and after the introduction of RDTs. It also measured the perceptions and usefulness of RDTs before their introduction and assessed the problems that health workers had with using RDTs to diagnose malaria after the tests were introduced.

## Methods

### Study Area

The study was undertaken in the Enugu East Local Government Area (LGA) in Enugu State, southeast Nigeria. The Enugu East LGA had a population of 279,089 in 2006 [25]. It has 12 public health centres, and 30 private clinics and hospitals. The health centres are stratified into three groups with high, medium, and low levels of infrastructure based on the number of staff, availability of relevant facilities, such as maternity beds, and utilization rates. All the centres have drug-dispensing units but no laboratory facilities. The 4 health centers with high-level infrastructure were purposely selected for the study. This was to enhance the recruitment of patients, as malaria cases were more likely to be seen there than in the low- or medium-level health centers. There is a high transmission rate of malaria year-round in the study area, with an average monthly malaria incidence of 6% [26].

### Study Design

The study was conducted from 2005 to 2008 as a component of a larger study that lasted for 30 months [27]. This component of the study compared the prescription practices of health workers before the introduction of the RDTs in the larger study and 12 months after their introduction in 4 health centers. The study also examined the perceptions of health workers towards different diagnostic methods before the introduction of RDTs. The study was conducted when ACTs were newly introduced in the country as a first-line antimalarial drug as a result of extensive resistance to chloroquine and sulphadoxine-pyrimethamine (SP), and following WHO's recommendation that a combination of antimalarials be used to treat malaria caused by P. falciparum. The drug resistance level to SP and chloroquine at the time of the study was more than 70% [1].

### Introduction of RDTs and ACTs

Before the introduction of RDTs and ACTs, as a supplement to the manufacturer's instruction, the health workers in the larger study were trained for three days by the research team on how to use RDTs and read the results. Parasitological tests for malaria were then undertaken using an RDT-ICT Malaria Combo Cassette Test (ML02) (ICT Diagnostics, Cape Town, South Africa). The sensitivity and specificity of the test in another setting have been found to be 96% and 95%, respectively [28]. The researchers observed how the health workers performed and interpreted the tests by paying them visits twice every week for 2 months. Correction was provided where it was observed that the health workers were having trouble with the products. At the same time, an ACT (dihydroxy-artemisinin/piperaquine) was introduced to complement the RDTs.

### Data Collection

All the 32 health workers in the 4 health centers were interviewed 12 months after the introduction of RDTs. Through open-ended questions, information was sought on their perceptions of symptoms-based, RDT-based, and microscopy-based malaria diagnoses, and how these have affected their diagnosis and treatment of malaria. Information was also collected on the problems they encountered while using the test, and its perceived effectiveness (ease of use) and diagnostic accuracy. They were also asked to suggest how RDT use by health workers could be improved. Although some health facilities did not have microscopes, all the health workers knew about microscopy for malaria diagnosis and may have been exposed to it in the past.

For the prescription analysis, the sample size was determined according to the WHO manual on how to investigate drug use in health facilities [29]. In addition to following the WHO-recommended sample size of a minimum of 20 prescriptions per facility, we decided to increase the number of prescriptions per facility to 50 to control for the design effect of clustering of multiple patients seen by the same health worker. Thus, a total of 200 prescriptions before and 200 after the RDT introduction were collected, observed, and recorded. The prescription analysis was only for the first visit and no re-attendances were taken.

Therefore, in each of the four health centers the average number of drugs (any drugs) per prescription, and the percentage of prescriptions with antibiotics, injections (any injections), and 3 antimalarial drugs (ACT, SP, and chloroquine) were determined. Also, data on the number of RDT-negative and RDT-positive patients who received ACTs were collected.

Before the RDT introduction, the prescription data were collected from the outpatient cards of the last 50 patients who presented with fever. After the RDT introduction, the prescription and diagnostic data were collected from the outpatient cards of the last 50 patients who presented with fever and which contained RDT results. The cards containing RDT results and the prescriptions given were routinely retained at each facility. The prescription data were collected before the introduction of RDT and 12 months thereafter to allow enough time to elapse so as to obtain reliable information about the impact, influence, and perception of RDTs from health workers, as it may not have been possible to get accurate information if the evaluation was made within a short period.

### Data Analysis

Data entry was done using Epi Info version 3.5, while analysis was done using SPSS version 11. The frequency distribution of the health workers' responses was computed. The data on prescribing patterns in the pre- and post-RDT introduction periods were compared. The key variables that were compared between the two periods were: the average number of drugs (any drugs) prescribed per encounter, the average number and type of antimalarials prescribed per encounter, the average percentage of prescriptions with one or more antibiotics, and the average percentage of prescriptions with any injections. In addition, the number of RDT-negative and RDT-positive cases that received ACTs as well as the non-antimalarial drugs prescribed for RDT-positive and RDT-negative results were analysed. Student's t-test was used to analyse continuous variables and the chi-square test for categorical variables. All tests of significance were done based on a p-level of 0.05. The design effect was accounted for in the final statistical analyses by multilevel modeling.

### Ethical Aspects

This research was approved by the Medical Research Ethics Committee, University of Nigeria Teaching Hospital, Enugu. Individual written and signed informed consent was obtained from all participants following verbal and written explanations of the study aims and procedures.

## Results

### Characteristics of the respondents

As shown in table 1, most of the respondents 30 (93.7%) were females. Nineteen (59.4%) of the health workers were community health extension workers, 8 (25%) were staff nurses/midwives, and only 4 (12.5%) and 1 (3.1%), respectively, were pharmacy technicians and a doctor. The majority of the respondents, 27 (84.4%), had been working in the health centers for more than a year and therefore were there when the RDT was introduced. Twenty-eight (87.5%) respondents had received RDT training.

**Table 1:** Socio-demographic characteristics of the respondents.

Variables	N = 32n (%)
**Sex**MaleFemale	2 (6.3)30 (93.7)
**Cadre**Staff nurse/midwifeCommunity Health extension workerPharmacy technicianDoctor	8 (25.0)19 (59.4)4 (12.5)1 (3.1)
**Length of time worked in the facility**0–1 yearAbove 1 year	5 (15.6)27 (84.4)
**Whether trained in the use of RDTs**YesNo	28 (87.5)4 (12.5)

### Health workers' perceptions of different diagnostic methods

Table 2 shows that the majority of the health workers (21, 65.6%) were of the opinion that the RDT is more effective for malaria diagnosis than microscopy (8, 25.0%) and clinical diagnosis (3, 9.4%). They also felt that the benefits of RDTs included increased use of RDTs in the facilities (24, 75.0%) and the tendency to prescribe more ACTs (25, 78.1%) and less chloroquine (13, 40.6%). All the health workers felt that RDTs led to a fast diagnosis.

**Table 2:** Respondents' perception of different diagnostic methods.

Variables	N = 32n (%)
**Perceived effectiveness of different diagnostic methods (accuracy and ease of use)**RDTClinical diagnosisMicroscopy	21 (65.6)3 (9.4)8 (25.0)
**Benefits of RDT**Increased use of RDTPrescribes more ACTPrescribes less chloroquineFast diagnosis	24 (75.0)25 (78.1)13 (40.6)32 (100)
**Problems encountered**Difficulty in drawing and collecting finger stick blood samplesDifficulty with transferring the blood to the test deviceReading test results too soon (before 15 minutes had elapsed)Misinterpreting faint positive test lines as negativeUnsafe handling and disposal of sharpsNot very happy when the test showed nomalaria if they believed that patient had it	10 (31.3)8 (25.0)5 (15.6)8 (25.0)2 (6.3)17 (53.1)
**Suggestion for improved RDT use at the health centers**More trainingProvision of pictorial job aidNeed to always look at the instructions to remind one of certain stepsPerform a repeat test when the result is negative	22 (68.8)27 (84.4)17 (53.1)9 (28.1)

### Use of RDTs by health workers

Some of the health workers experienced difficulties in the process of using the RDT kits. These problems included difficulty in collecting blood from the finger and transferring it to the test device, and in timing the duration of the test before the results are read. Misinterpreting faint positive test lines as negative, and unsafe handling and disposal of sharps also posed challenges to some of the health workers. To improve their use of RDTs in the facilities, the health workers suggested additional training on RDT use, the provision of pictorial job aids, reminders to always look at the instructions to prompt oneself about certain steps, and performing a repeat test when the result is negative.

### Antimalarial prescription practices of health workers

As shown in table 3, the average numbers of drugs per prescription were 6.2 and 3.3, respectively, for the period before and after the RDT introduction (p<0.05). The average percentages of prescriptions with injections were 43.5 and 11.5%, respectively, for the period before and after the RDT introduction (p<0.05). Also, the average percentages of prescriptions with one or more antibiotics were 75 and 62%, respectively, for the period before and after the RDT introduction (p<0.05).

**Table 3:** Prescription practices of health workers before and after RDT introduction.

Indicators	Before RDT	After RDT	P value
Average number of any drugs per prescription (SD)	6.2 (2.4)	3.3 (1.8)	0.00004
Average percentage of prescriptions with any injections	43.5%	11.5%	0.0001
Average percentage of prescriptions with one or two antibiotics	75%	62.0%	0.0478
Average percentage of prescriptions with ACT	1.5%	86.0%	0.0001
Average percentage of prescriptions with SP	19.5.0%	2.5%	0.0001
Average percentage of prescriptions with Chloroquine	79.0%	11.5%	0.0001

Also, in the period before the RDT introduction, the average percentages of prescriptions with ACT, SP, and chloroquine were 1.5, 19.5, and 79%, respectively, compared with 86.0, 2.5, and 11.5%, respectively, in the post-RDT era. There were statistically significant differences in SP, chloroquine, and ACT prescriptions between the pre- and post-RDT eras.

Table 4 shows that in the post-RDT intervention period, a total of 92 (46.0%) prescriptions contained RDT-positive results and 108 (54.0%) had RDT-negative results. All the RDT-positive results were prescribed ACTs. However, of the RDT-negative results, 80 (74.0%) were prescribed ACTs. On the whole, a total of 172 (86%) patient cards had ACT prescriptions.

**Table 4:** ACT prescription practices of health workers after RDT introduction.

Indicators	N (%)
RDT ResultsPositiveNegativeTotal	92 (46.0)108 (54.0)200 (100.0)
ACT Prescription for RDT positive resultsYesNoTotal	92 (100.0)0 (0.0)92 (100.0)
ACT Prescription for RDT negative resultsYesNoTotal	80 (74.0)28 (26.4)108 (100.0)

### Prescribing of non-antimalarial drugs by RDT test results

As shown in table 5, in the post-RDT intervention period, when the prescribing of non-antimalarial drugs was stratified by RDT test results, the numbers of drugs per prescription for those with RDT-positive and RDT-negative results were 2.1 and 3.8, respectively. Also, the average percentages of RDT-positive and RDT-negative prescriptions with injections were 17.4% and 82.6%, respectively. The average percentages of RDT-positive and RDT-negative prescriptions with one or two antibiotics were 7.3 and 92.7%, respectively. The differences in prescription between RDT-positive and RDT-negative results were statistically significant.

**Table 5:** Prescribing of non-antimalarial drugs by RDT test results.

Indicators	RDT Positive	RDT Negative	P value
Average number of drugs per prescription (SD)	2.1 (1.1)	3.8 (1.5)	0.0084
Average percentage of prescriptions with injections. n (%)N = 23	4 (17.4)	19 (82.6)	0.0001
Average percentage of prescriptions with one or two antibiotics. n (%)N = 124	9 (7.3)	115 (92.7)	0.0001

## Discussion

The introduction of RDTs led to a reduction in the prescription of antimalarial drugs (chloroquine and SP). Conversely, the prescription of ACTs increased after the introduction of RDTs. However, health center workers gave ACTs to patients with negative RDT results and this was quite high. Studies [8], [30] in other settings have also confirmed this trend in which clinicians were reluctant to refrain from treating malaria even after a negative RDT test. In another study, 80 to 85% of RDT-negative febrile patients were treated for malaria [31], while still another study reported a level as low as 16% [17]. This non-compliance with the test results in our study may be associated with the fact that 12 months after the introduction of RDTs in the study area, no effort had been made by the government to monitor and supervise the health workers on the use of RDTs, except for the supervision done by the research team in the first two months. Thus, the initial zeal to adhere to RDT results experienced in the early phase of the RDT introduction may have waned.

Not all the health workers agreed that the benefit of RDTs included increased usage because making RDTs available does not always translate to use, as some may have felt it was not necessary. The use of RDT is expected to reduce the overuse of antimalarial drugs, especially the expensive ACTs, by ensuring that treatment is targeted to patients suffering from malaria as opposed to treating all patients with fever, which was the case when chloroquine was the first-line drug. It has been noted that health workers' adherence to a test-based strategy is a key factor in determining whether the strategy is effective in improving management and curtailing costs [32], [33]; the argument has been that, if the result of a test is not going to influence management, then doing the test is a waste of money [32]. Therefore, there is a need for the government to engage in supportive supervision of health workers and to constantly remind them that the cost of treating all individuals regardless of the test results is enormous, both for the patient and to the health services.

In contrast to our results, high adherence to RDT results by health workers was reported in Tanzania [34] and Zanzibar [23]. However, in the Zanzibar study, supervision and incentives were provided to the nurses, which may have resulted in the high adherence. Also, the prescribers themselves were the research assistants and thus had to record their own prescribing behaviour, which may have influenced their decisions. In addition, the assessments in these studies were done immediately after the intervention, in contrast to our assessment, which was carried out 12 months after the RDT introduction. Thus, with the lack of supervision and incentives in our study area, the health workers were unlikely to adhere to RDT-negative results. Interestingly, the risk of a false-negative test and its potential consequences have recently been evaluated in Uganda [35] and Tanzania [36], and the safety of not treating malaria-negative children was confirmed.

If RDTs are to be effective in malaria programmes, the need to manage RDT-negative results should be addressed; otherwise, health workers will continue to treat many cases of non-malarial fever with ACTs, and the potential benefit of malaria RDTs in improving the early management of non-malarial febrile illness through early diagnosis and exclusion of malaria as a cause would be lost. One option is to develop and provide management algorithms for the appropriate management of RDT-negative cases and to train health workers on their use. This algorithm should include a pathway that will enable the health workers to always perform a repeat test when the result is negative if they strongly feel that the patient has malaria and a pathway for the appropriate referral and follow-up of RDT-negative patients. The community members should be empowered through appropriate sensitization on the importance of parasite-based diagnosis and the need to insist on taking ACTs only when the RDT result is positive. However, changing the behaviour of health workers on this matter has presented a major challenge to the program in Madagascar [37].

The results of this study indicate that health center workers perceive the RDT to be more effective for malaria diagnosis than microscopy and clinical diagnosis, and that it has a lot of benefits. This is despite the fact that microscopy is regarded as the gold standard for malaria diagnosis. This is a positive development, as this shows the acceptability of RDTs among the health workers. Although the reasons for this perception were not explored, the fact that RDTs were readily available, the results were known within a short time, and treatment was given immediately may have contributed to this perception.

The health workers identified some potential concerns regarding the use of RDTs. The first relates to the technique of performing the finger prick, and collecting and transferring blood to the test device. Although health workers are used to collecting blood from patients with needles and syringes for other laboratory investigations, most of them had never taken a finger prick blood sample before the study and had some difficulties with their initial attempts. Some of them claimed that at times they stab too lightly and at other times too deeply. In both cases, there is a tendency to collect too little or too much blood. It has been noted that an inadequate blood volume can reduce sensitivity, while an excessive volume may cause background staining and obscure faint results [38]. Collecting too little blood might also cause the health workers to repeat the finger prick, which might scare patients away. In addition, some of the health workers said they read the test results too soon. This problem has been noted elsewhere [39], and it has been suggested that the reason for this might be that the package instructions give insufficient emphasis to the importance of waiting. Hence, the RDT manufacturer might do well to lay emphasis on the waiting time.

Another concern raised by the health workers was the incorrect interpretation of test results; they said that on some occasions, they read faint positive or invalid tests as negative. It is known that the strength of the test line can vary significantly depending on the level of parasitaemia, blood viscosity, volume of blood, and other factors [40]. The inability to correctly interpret this might be due to improper training, absence or lack of training (as some of the health workers were posted to the health centers after the introduction of RDTs), or visual acuity problems. Training health workers, and any users of RDTs in the community for that matter, to recognize faint results is likely to enhance their performance and confidence in reading the test results. The results also show that health workers are usually not very happy when the test shows no malaria, especially if they believe the patient has the disease, as they are likely to lose a client. This was buttressed by the results of the prescription analysis, which showed excessive prescription of ACTs in RDT-negative results. Evidence has shown that most of the illnesses treated in health centers in Nigeria are due to presumptive malaria [41].

The smaller number of drugs, antibiotics, and injections per prescription post-RDT intervention may be a reflection of an increased knowledge of prescribing practices among health workers as a result of the evidence of the presence or absence of malaria produced by RDTs and the availability of ACTs in the facilities. Polypharmacy is likely to be encouraged by the absence of a diagnostic method. In Nigeria evidence exists that health personnel tend to engage in polypharmacy in their attempts to treat a number of possible diseases simultaneously in the absence of a definitive diagnosis [42]. In some settings the prescription rate of antibiotics was found to be higher after RDTs were introduced; the authors have suggested that RDT-negative results led nurses to consider and treat alternative causes of fever [17]. The reverse was the case in our study, suggesting that the health workers may have been restrained from engaging in polypharmacy, in which providers prescribe additional unnecessary drugs, and may have considered the overprescription of ACTs in the event of RDT-negative results. In both cases, the prescription was irrational and the losses from irrational drug prescriptions have been estimated to reduce drug availability by 50% [29]. However, in the pre- and post-evaluation periods of this study, all the assessed drugs were present in the facilities, although in different quantities; this difference may have also affected the prescriptions.

We acknowledge that there may have been some bias in the results due to historical evolution or concurrent unknown interventions that took place in the study area in the intervening period. Hence, because there were no comparative data, it would not have been possible to detect the occurrence and effects of such concurrent unknown interventions. However, the authors were not aware of any such intervention with RDT after our intervention in the study area.

### Conclusions

The introduction of RDTs increased the prescription of ACTs but equally increased its overprescription, with the drug being prescribed to people with RDT-negative results. However, this conclusion is tempered by the fact that the study had no control arm to detect the effects of other concurrent interventions. Therefore, in order to improve rational prescribing and tap the gains of the concurrent introduction of ACTs and RDTs, the overprescription of ACTs should be tackled by encouraging health workers to trust the RDT results and prescribe ACTs only to those with positive RDT results. Because the health workers may have become so accustomed to the clinical approach to malaria diagnosis, there is a need to remind them regularly of the potential savings and reductions in suffering that come from more rational antimalarial drug use. Malaria programme officers and national malaria control programmes will therefore need to follow up on the training and retraining of health workers on the use of RDTs and correct some of the problems the health workers encountered while using RDTs.
